# HSP90 overexpression potentiates the B-cell receptor and fibroblast growth factor receptor survival signals in chronic lymphocytic leukemia cells

**DOI:** 10.18632/oncotarget.27409

**Published:** 2020-06-02

**Authors:** Hasan Mahmud, Mariana Mendez, Bedabrata Mukhopadhyay, Jennifer Holter-Chakrabarty, Asish K. Ghosh

**Affiliations:** ^1^Stephenson Cancer Center, University of Oklahoma Health Sciences Center, Oklahoma City, OK 73104, USA; ^2^Department of Pathology, University of Oklahoma Health Sciences Center, Oklahoma City, OK 73104, USA

**Keywords:** CLL, HSP90, BCR, CD79a, PTPN22

## Abstract

Chronic lymphocytic leukemia (CLL) is still an incurable disease despite aggressive chemotherapies including the B-cell receptor (BCR) targeted-inhibitors. Therefore, we assessed the expression status of key signal mediators of the BCR pathway in CLL cells. Indeed, we detected aberrantly elevated levels of CD79a, B-cell adaptor for PI3K (BCAP) and phospholipase C (PLC)γ2, key mediators of BCR signal, in CLL cells. As HSP90 is also overexpressed in CLL cells, we hypothesized that HSP90 could potentiate the BCR signal via stabilization of multiple key components of the BCR-signalosome. We found that HSP90 formed a multi-molecular complex with CD79a, BCAP, PLCγ2, LYN, SYK, Bruton tyrosine kinase (BTK) and AKT and that, pharmacologic inhibition or partial depletion of HSP90 reduced the expression of these signal mediators in CLL cells. In addition, our findings also demonstrated that HSP90 could stabilize the tyrosine phosphatase, PTPN22 which positively regulates AKT phosphorylation, and the constitutively active fibroblast growth factor receptor 3 (FGFR3) in CLL cells. Finally, HSP90 inhibition induced apoptosis in CLL cells in a dose-dependent manner likely via downregulation of anti-apoptotic proteins MCL-1 and XIAP, but not BCL2, reported to be overexpressed in CLL cells. In total, our findings suggest that HSP90-inhibition may sensitize the leukemic B-cells to BCR-targeted agents, particularly those become resistant to these therapies.

## INTRODUCTION

CLL is the most common form of adult leukemia in the Western hemisphere [1]. It is characterized by the accumulation of CD19^+^/CD5^+^/CD23^+^ mature, monoclonal B-cells in the blood, bone marrow and lymphoid tissues. Although introduction of BCR-targeted therapies has remarkably changed the landscape of CLL management, long-term disease control or prevention of relapse is still not achieved [2].

BCR signaling pathway has emerged as a key driver for the expansion of neoplastic B-cell clones and pathogenesis in several B-cell malignancies including CLL [3]. Activation of BCR induces phosphorylation at the tyrosine residues in the immunereceptor tyrosine-based activation motif (ITAM) of a heterodimer CD79a/CD79b (which remain in a complex with a membrane immunoglobulin) via the Src family kinase LYN and spleen tyrosine kinase (SYK). Phosphorylation of the ITAM results in recruitment of the signalosome which consists of kinases, adaptor proteins, lipase, lipid kinase and phosphatases including LYN, SYK, BTK, PLCγ2, PI3Kδ, BCAP and induces activation of a cascade of downstream signaling pathways: AKT, NF-κB and ERK1/2. While the use of BCR-targeted oral agents ibrutinib (BTK-inhibitor) and idelalisib (PI3Kδ-inhibitor) has been shown to be effective in relapsed/refractory CLL patients, the responses are limited to partial remissions [4]. However, when patients relapse, there is often evidence for more aggressive disease including transformation to diffuse large B-cell lymphoma (Richter’s syndrome) [5] which is difficult to treat. Currently, the only approved novel inhibitor strategies that may be of assistance when ibrutinib is unable to be used are idelalisib with rituximab or a BCL2-inhibitor, venetoclax [6]. However, significant toxicities have been reported for the regimen of idelalisib/rituximab with excess infectious deaths [7] and increased risk of immune based hepatic toxicity for CLL patients treated in an upfront setting while, with venetoclax, the risk of tumor lysis syndrome continues even with the use of a slow dose escalation. Therefore, insights into the unique mechanism(s) of BCR regulation and/or other cell survival pathways in CLL clones are critical to overcome resistance of the CLL patients to BCR-targeted agents. In this study, we have investigated if overexpression of critical signaling components of the BCR pathway potentiates BCR signal in CLL cells.

## RESULTS

### CLL cells overexpress CD79a, BCAP, PLCγ2 and HSP90

**Figure 1:**
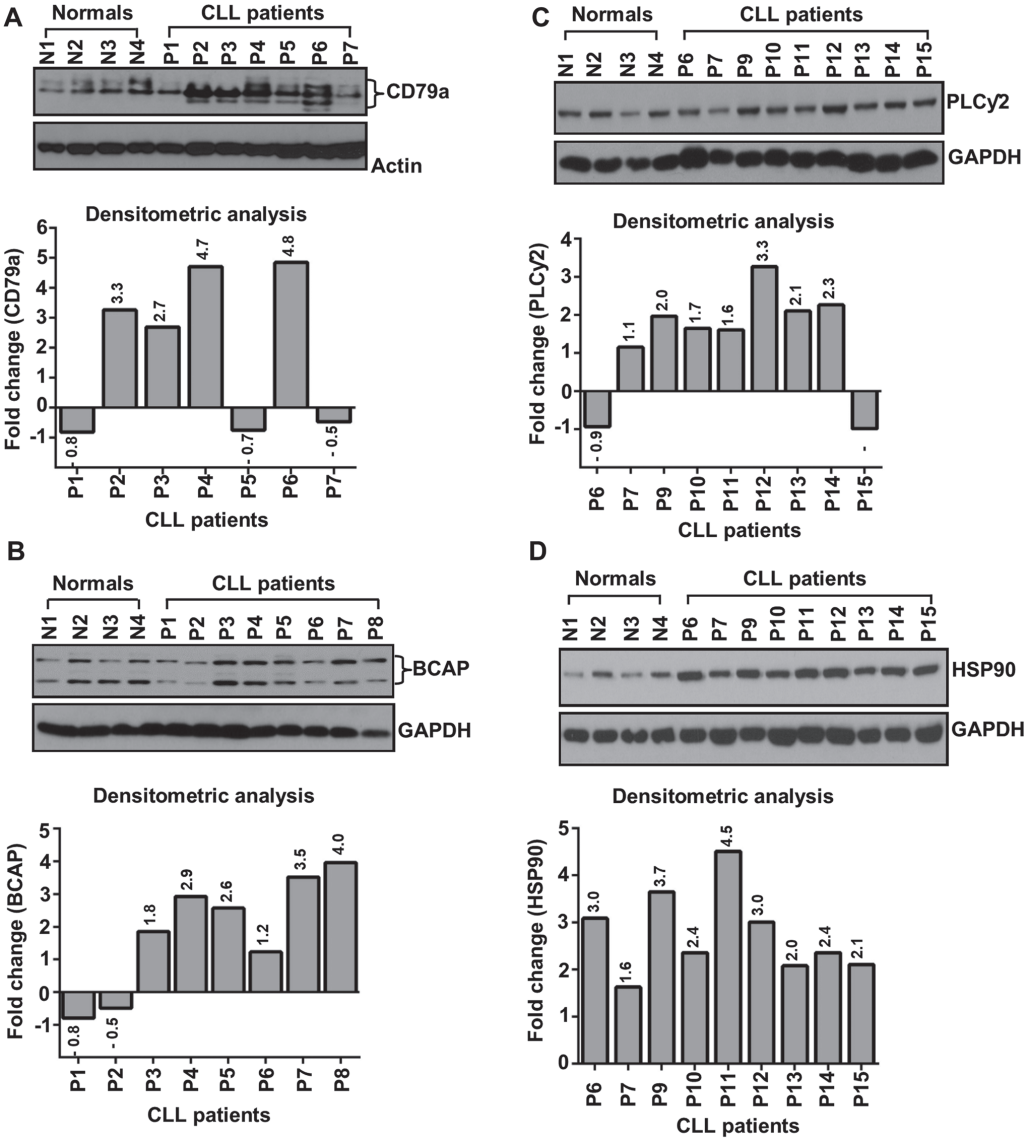
CLL cells aberrantly express CD79a, BCAP, PLCγ2 and HSP90. Lysates from purified normal B-cells and CLL cells were analyzed for the expression of CD79a (**A**), BCAP (**B**), PLCγ2 (**C**) and HSP90 (**D**) in western blots using specific antibodies. Actin or GAPDH was used as loading control. Expression levels of specific proteins were quantified by densitometric analyses (target protein: loading control) and presented as “fold changes” with respect to those in normal B-cells (bottom panels). Normal subjects (N1 – N4) and CLL patients (P1 – P15) are indicated by assigning numbers.

Lysates from purified, normal B-cells or CLL cells from previously untreated CLL patients were examined for the expression of CD79a, BCAP and PLCγ2 in western blots using specific antibodies. Results obtained were quantified by densitometric analysis. While earlier work reported overexpression of multiple BCR downstream signal mediators including BTK [8], LYN [9] and SYK [10], here, we have detected aberrant expression of CD79a (Figure 1A), BCAP (Figure 1B) and PLCγ2 (Figure 1C) in CLL cells from majority of previously untreated CLL patients independent of their prognostic parameters (Supplementary Table 1) as compared to normal B-cells. However, the regulation of CD79a, BCAP or PLCγ2 overexpression in CLL cells remains unknown.

Heat shock protein 90 (HSP90) is an ATP-dependent molecular chaperon that ensures the correct folding and stability of more than 200 “client” proteins, many of which are oncoproteins including kinases and transcription factors [11], and remains aberrantly expressed in many human cancers including CLL [12]. In the absence of HSP90 binding, rapid degradation of its client proteins occurs via the proteasomal system. Therefore, we hypothesized that overexpression of HSP90 regulates the BCR signaling pathway in CLL cells via stabilization of multiple signal mediators. To address this, we further assessed HSP90 expression status in CLL cells by western blot analysis. As reported earlier [12], we also detected aberrantly elevated levels of HSP90 in CLL cells as compared to normal B-cells (Figure 1D).

### Pharmacologic inhibition or partial depletion of HSP90 alters the expression levels of BCR signal mediators

**Figure 2:**
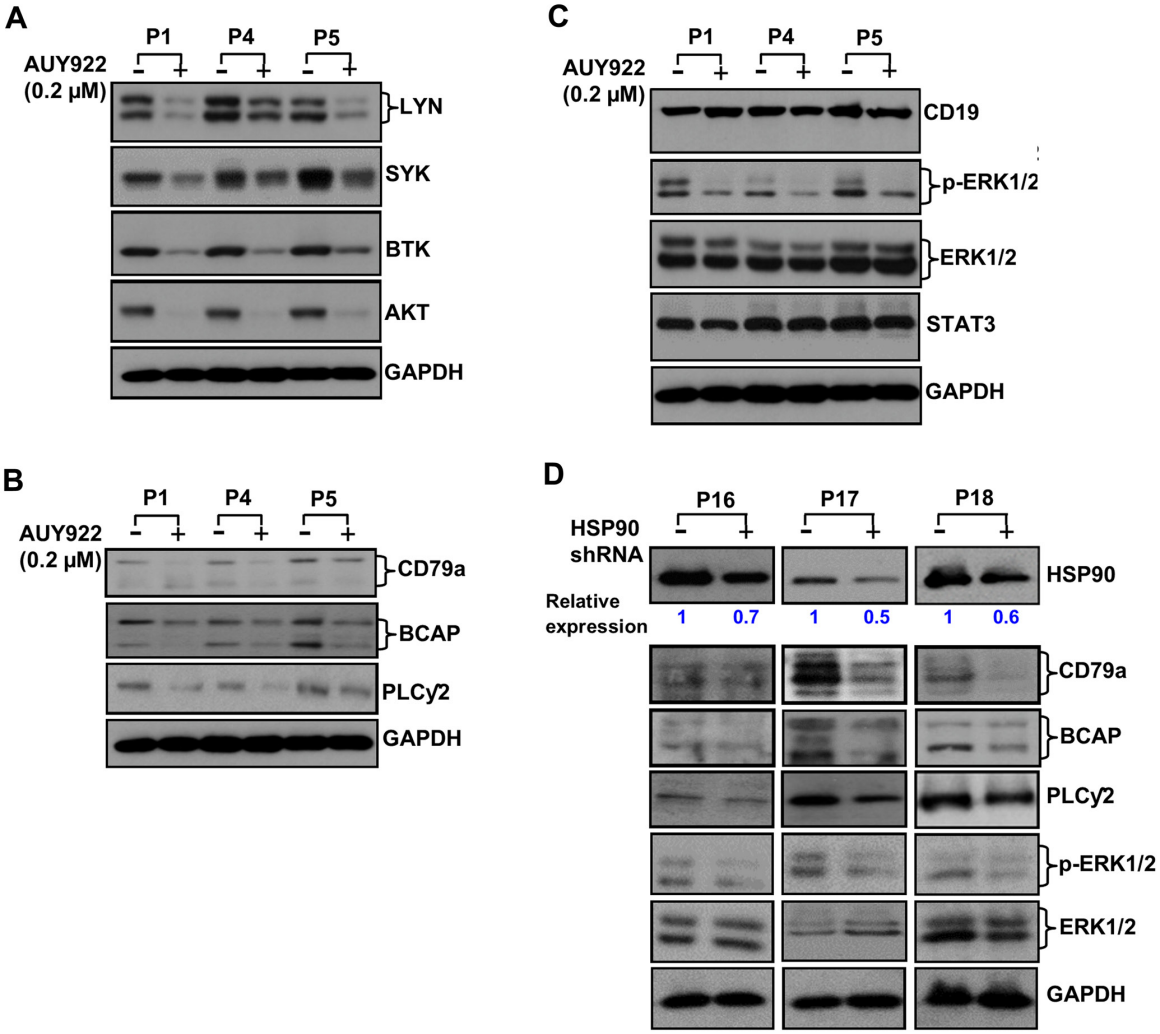
Pharmacologic inhibition or partial depletion of HSP90 in CLL cells reduces the levels of BCR signal mediators. (**A**–**C**) Impact of HSP90 inhibition on BCR signal mediators. Purified CLL cells were treated with a high-affinity HSP90 inhibitor, AUY922, at 0.2 µM dose or DMSO (vehicle control) for 24 hours. Cell lysates were analyzed for the expression of various signal mediators of the BCR pathway in western blots using specific antibody to LYN, SYK, BTK or AKT (A). Expression status of CD79a, BCAP, and PLCγ2 was also analyzed in the same CLL cell lysates used above in western blots using specific antibodies (B). Endogenous expression levels of CD19, ERK1/2 or STAT3 which are not reported to be the HSP90 client proteins were also analyzed in the same CLL cell lysates used above (C). However, phosphorylation status of ERK1/2 was examined as a downstream target of BCR signal in these cell lysates (C). GAPDH was used as loading control. CLL patients (P1, P4, P5) are indicated by numbers. (**D**) Targeted depletion of HSP90 reduces the levels of CD79a, BCAP, PLCγ2 and phosphorylation of ERK1/2 in CLL cells. Purified CLL cells from three previously untreated CLL patients (P16 – P18) were transduced with lentivirus expressing a HSP90-specific shRNA (indicated by “+”) or scrambled shRNA (indicated by “-”) for 24 hours. Cell lysates were prepared and analyzed for the expression of HSP90, CD79a, BCAP, PLCγ2 and P-ERK1/2 in western blots using specific antibodies. The blot of P-ERK1/2 was stripped and reprobed with an antibody to total ERK1/2. GAPDH was used as loading control. Level of HSP90 depletion in HSP90-shRNA transduced CLL cells was determined by densitometric quantification and presented as relative values of HSP90: GAPDH with respective to the scrambled shRNA transduced cells.

To serve as a chaperone protein, HSP90 has to be in an active conformation, which is commonly seen in transformed but not in normal cells. Therefore, to address the hypothesis that overexpressed HSP90 stabilizes the BCR signalosome, we inhibited HSP90 in CLL cells using a high-affinity HSP90-inhibitor, AUY922 [13]. Indeed, a significant reduction of LYN, SYK, BTK and AKT protein levels (Figure 2A) was discernible in CLL cells upon HSP90-inhibition as reported recently [14], while our work was in progress. As a result of such substantial reduction of LYN/SYK/BTK/AKT, we anticipated that constitutive levels of phosphorylation on these signal mediators would also be reduced in CLL cells upon AUY922 treatment. As expected, activating phosphorylation (Y397) level of LYN was significantly reduced in CLL cells treated with AUY922 (Supplementary Figure 1). Interestingly, HSP90-inhibition in CLL cells also reduced the protein levels of aberrantly elevated CD79a, BCAP and PLCγ2 (Figure 2B). Although the expression of CD19, ERK1/2 or STAT3, known to be the HSP90 non-client proteins, remained unaltered upon HSP90-inhibition (Figure 2C), phosphorylation of ERK1/2 was significantly reduced in CLL cells likely, as a result of interruption of the BCR signal. Further, HSP90 was targeted by transducing CLL cells with lentivirus expressing scrambled or a HSP90-specific shRNA to rule out the possibility that the above findings were not just the off-target effects of AUY922. Indeed, partial depletion of HSP90 in CLL cells resulted in reduced expression of CD79a, BCAP and PLCγ2 with significant inhibition of phosphorylation on ERK1/2 but not the total ERK1/2 protein levels (Figure 2D). These results corroborated well with the findings of pharmacologic inhibition of HSP90 by AUY922 in CLL cells (Figure 2B and 2C). In total, these observations suggest that aberrant increase of HSP90 in CLL cells may potentiate the BCR signal likely by chaperoning multiple critical components of the BCR pathway including CD79a, BCAP, PLCγ2, LYN, SYK, BTK and AKT from proteosomal degradation.

### HSP90 forms multi-molecular complex with BCR signal mediators in CLL cells

**Figure 3:**
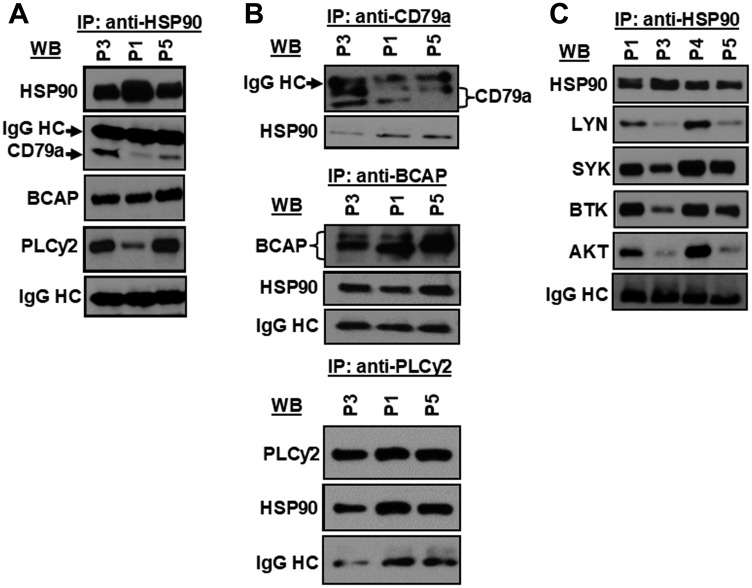
HSP90 forms a multi-molecular complex with kinases, lipase and adaptor molecule of the BCR pathway. HSP90 was immunoprecipitated from CLL cell lysates from previously untreated CLL patients (P1, P3, P5), followed by detection of CD79a, BCAP, and PLCγ2 (**A**) in western blots using specific antibodies. Similarly, CD79a, BCAP or PLCγ2 was immunoprecipitated individually from the same CLL cell lysates (P1, P3, P5) used above and the immunecomplex was analyzed for the presence of HSP90 in western blot using a specific antibody to HSP90 (**B**). HSP90 was further pulled down from the same CLL cell lysates (P1, P3, P5) used above to detect co-precipitation of LYN, SYK, BTK or AKT by western blot analyses using specific antibodies (**C**). CLL B-cell lysates from another CLL patient (P4) was also included in panel C. Immunoglobulin G heavy chain (IgG HC) was used as loading control.

Next, to interrogate if CD79a, BCAP or PLCγ2 were indeed the targets of the HSP90 chaperon complex, HSP90 was immunoprecipitated from CLL cell lysates using an antibody to HSP90_αβ_ (Santa Cruz), Protein G-agarose beads (Cell Signaling Technologies) alone or isotype control antibody (IgG2a; Cell Signaling). We found that CD79a, BCAP and PLCγ2 were also co-immunoprecipitated with HSP90 from CLL cell lysates when the immunecomplex was analyzed in western blots using specific antibodies (Figure 3A). However, HSP90 was not detected in the immunecomplex of the control antibody or agarose beads in western blot analyses (Supplementary Figure 2). A reverse immunoprecipitation assay using the same CLL cell lysates further confirmed that CD79a, BCAP and PLCγ2 indeed formed a complex with HSP90 in CLL cells (Figure 3B). In addition, we also detected that LYN, SYK, BTK and AKT were in the same HSP90-immunecomplex (Figure 3C) analyzed in Figure 3A. Together, these findings suggest that HSP90 may regulate the BCR signal in CLL cells via stabilization of multiple components of the BCR signalosome and that, CD79a, BCAP and PLCγ2 are the new addition to the growing “client-protein” list of HSP90.

### CLL cells overexpress PTPN22

**Figure 4:**
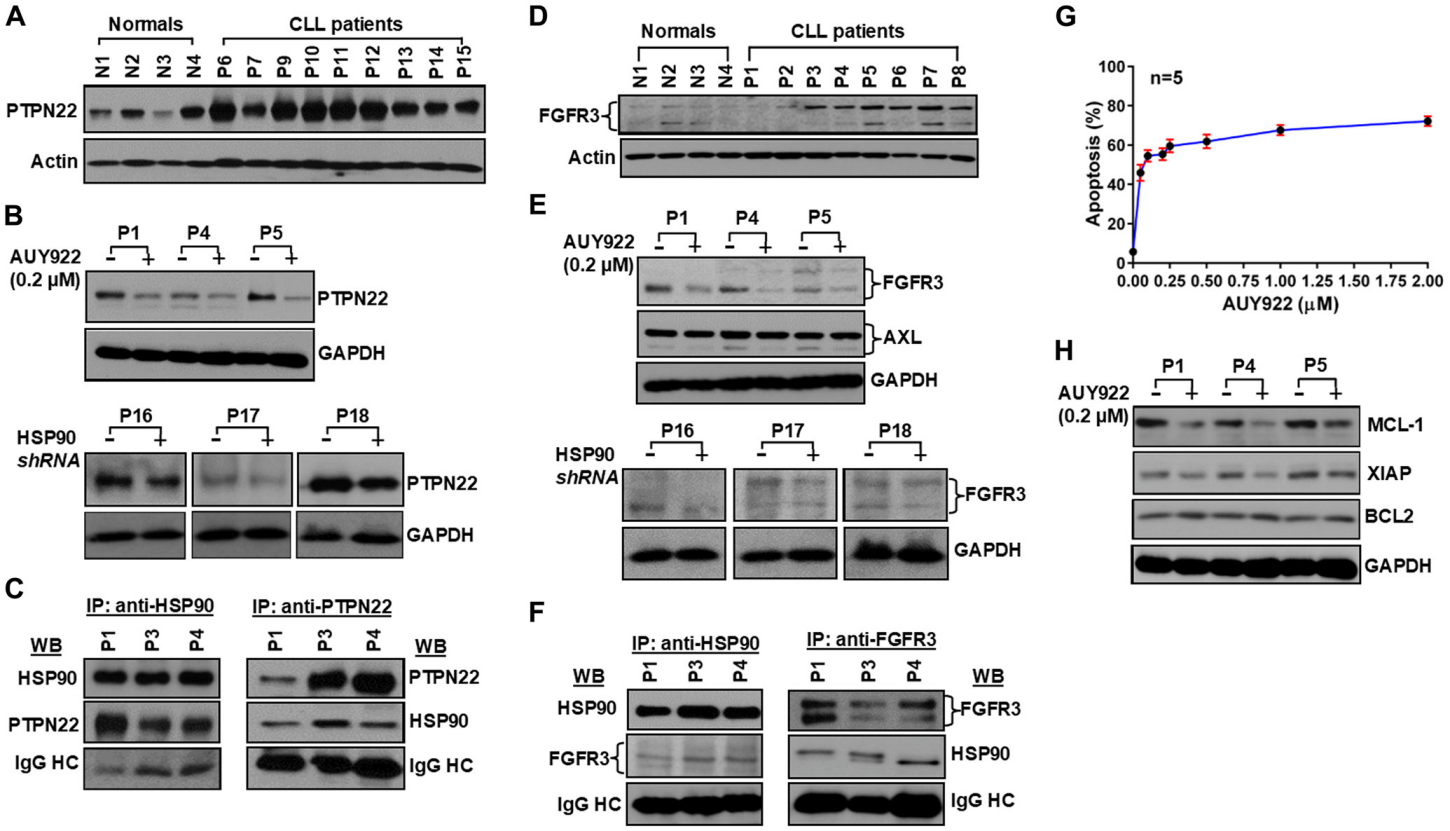
Aberrantly expressed PTPN22 and FGFR3 are client proteins of HSP90, and pharmacologic inhibition of HSP90 induces apoptosis in CLL cells. (**A**) CLL cells overexpress PTPN22. Lysates from purified normal B-cells and CLL cells were analyzed for the expression of PTPN22 in western blot using a specific anti- PTPN22 antibody. Actin was used as loading control. Normal subjects (N1–N4) and CLL patients (P6, P7, P9–P15) are indicated by assigning numbers. (**B**) Pharmacologic inhibition or partial depletion of HSP90 decreases PTPN22 expression. Lysates of CLL cells treated with AUY922 (upper panel; P1, P4, P5) or transduced with lentivirus expressing scrambled or HSP90-specific shRNA (lower panel; P16–P18) were analyzed for the expression of PTPN22 in western blots using specific antibody. GAPDH was used as loading control. (**C**) HSP90 forms complex with PTPN22. HSP90 (left panel) or PTPN22 (right panel) was immunoprecipitated from the same CLL cell lysates (P1, P3, P4) using specific antibody, followed by detection of PTPN22 (left panel) or HSP90 (right panel) respectively, in western blot analyses. The blots were stripped and reprobed to detect immunoprecipated proteins using specific antibodies. (**D**) CLL cells overexpress FGFR3. Lysates from normal B-cells and CLL cells used in Figure 1B were analyzed for the expression of FGFR3 in western blot using a specific anti- FGFR3 antibody. Actin was used as loading control. (**E**) Pharmacologic inhibition or partial depletion of HSP90 reduces FGFR3 protein levels. Lysates of CLL cells treated with AUY922 (upper panel; P1, P4, P5) or transduced with lentivirus expressing scrambled or HSP90-specific shRNA (lower panel; P16–P18) were analyzed for the expression of FGFR3 in western blots using specific anti- FGFR3 antibody. Expression of AXL was also analyzed in AUY922-treated CLL cell lysates using a specific antibody to AXL. GAPDH was used as loading control. Of note, CLL cell lysate of P17 used in panels B and E showing the same loading control, GAPDH. (**F**) HSP90 and FGFR3 form a complex in CLL cells. HSP90 (left panel) or FGFR3 (right panel) was immunoprecipitated from the same CLL cell lysates (P1, P3, P4) using specific antibody, followed by detection of FGFR3 (left panel) or HSP90 (right panel) respectively, in western blot analyses. The blots were stripped and reprobed to detect immunoprecipitated proteins using specific antibodies. (**G**) HSP90 inhibition induces apoptosis in CLL cells. Purified CLL cells from previously untreated CLL patients (*n* = 5) were treated with increasing doses of AUY922 (0.05–2 μM) for 72 hours and induction of apoptosis was determined by flow cytometric analysis after staining the cells with chromogen conjugated annexin V and propidium iodide. Results are presented as mean values ± standard deviations at each indicated dose. (**H**) HSP90 inhibition reduces the expression of anti-apoptotic proteins in CLL cells. Lysates of purified CLL cells (P1, P4, P5) treated with AUY922 used in panel 4B (upper blot) were further analyzed for the expression of MCL-1, XIAP and BCL2 in western blots using specific antibodies. The same loading control GAPDH was used for both the panels, 4B and 4H.

The intensity and duration of the BCR signal are controlled by various negative regulators, including inhibitory receptors, phosphatases and ubiquitin ligases. Importantly, some of these negative regulators are also activated by LYN, which functions as both a positive and negative regulator of the BCR signal. This dual role of LYN stems from its unique ability to phosphorylate the immunoreceptor tyrosine-based inhibitory motifs in the inhibitory receptors CD22, FcγRII, CD5 and CD72 [15]. Phosphorylation of these receptors brings the phosphatases SHP-1 and SHIP in the vicinity of the activated BCR, where they terminate the signal by dephosphorylating various activated components of the BCR signaling pathway [15] including phosphatidylinositol 3,4,5-triphosphate. The lymphoid phosphatase (LYP), a cytosolic protein tyrosine phosphatase encoded by the PTPN22 gene, has been shown to be overexpressed in CLL cells and positively regulates AKT signal downstream of BCR, at least in part, by reducing recruitment and activation of SHIP to the BCR [16]. Consistent with the earlier report [16], we have also detected PTPN22 overexpression in CLL cells as compared to normal B-cells (Figure 4A). While the regulation of PTPN22 overexpression in CLL cells was largely unknown, here, we found that targeted inhibition or partial depletion of HSP90 in CLL cells reduced PTPN22 protein levels (Figure 4B) when analyzed the same CLL cell lysates used in Figure 2. Furthermore, PTPN22 was also found to be physically associated with HSP90 (Figure 4C) as assessed by immunoprecipitation/western blot analysis. Thus, increased accumulation of PTPN22 in CLL cells may be explained, at least in part, by aberrant increase of HSP90 stabilizing the phosphatase via complex formation. In total, our findings suggest that HSP90 overexpression contributes significantly in regulating the BCR signal via formation of a multi-molecular complex with several BCR downstream signal mediators including kinases, adaptor (BCAP) and phosphatase (PTPN22) in CLL cells.

### HSP90 regulates FGFR signal in CLL cells

Despite a critical role of BCR signal in CLL cell proliferation and survival, CLL cells also overexpress multiple constitutively active receptor tyrosine kinases (RTKs) including AXL [17] and its downstream target, FGFR3 (Figure 4D) [18]. We have shown previously that AXL is ubiquitously expressed and constitutively active in CLL cells [17, 19], remains significantly elevated in cells with non-functional p53 [19] and regulates cell survival via activation of multiple downstream signal mediators. AXL/FGFR3 share common signal mediators with the BCR pathway including LYN, AKT and ERK1/2 to transmit survival signals [16–18]. However, the regulation of AXL or FGFR3 expression in CLL cells is largely undefined. To interrogate if AXL and FGFR3 are also regulated, at least in part, by HSP90, expression of both the RTKs was examined in CLL cells treated with AUY922 or transduced with a HSP90-targeted *shRNA*. Western blot analyses find a significant reduction of FGFR3 expression but not AXL (Figure 4E) upon pharmacologic inhibition (upper panel; P1, P4, P5) or *shRNA*-mediated partial depletion (lower panel; P16 – P18) of HSP90 in CLL cells. Furthermore, HSP90 forms a complex with FGFR3 (Figure 4F), but not with AXL (data not shown), in CLL cells. Thus, our results suggest that aberrantly expressed HSP90 not only regulates BCR signal in CLL cells, it may also regulate the FGFR-signal via stabilization of FGFR3 protein level.

### HSP90 inhibition induces apoptosis in CLL cells

Finally, we have shown that AUY922-mediated inhibition of HSP90 induces apoptosis in CLL cells in a dose-dependent manner (Figure 4G). Further analysis suggests that HSP90 inhibition results in downregulation of the anti-apoptotic proteins MCL-1 and XIAP, but not BCL2, in CLL cells (Figure 4H).

## DISCUSSION

Several lines of evidence support the hypothesis that CLL is a BCR-dependent malignancy [20–25]. Pro-survival signal generated by the BCR, transmitted through cytoplasmic tails of its CD79a and CD79b subunits, is aberrantly active in CLL cells and represents one of the most important oncogenic pathways in CLL involved in disease pathogenesis [26]. It is noteworthy that the expression of the CD79b subunit of BCR is reported to be down-regulated in CLL cells [27], suggesting that transmission of constitutive level of BCR signal may depend on the expression status of CD79a. In line of this agreement, we detected reduced protein levels of CD79b (Supplementary Figure 3) but increased expression of CD79a in CLL cells from majority of previously untreated CLL patients. In addition, we have also detected increased protein levels of BCAP and PLCγ2 in CLL cells, further indicating that stabilized accumulation of multiple signal mediators of the BCR pathway may promote a highly active and sustained BCR signal for prolonged survival of CLL cells which may also render the cells resistant to BCR-targeted therapies.

In this study, we have demonstrated that HSP90 is overexpressed in CLL cells that controls key signal mediators of the BCR pathway including CD79a, BCAP, PLCγ2, LYN, SYK, BTK and AKT. Of note, previous studies reported overexpression of LYN, SYK and BTK in CLL cells [8–10]. Indeed, our findings demonstrate that HSP90 remains in a multi-molecular complex with CD79a, BCAP, PLCγ2, LYN, SYK, BTK and AKT in CLL cells. Interestingly, HSP90 also forms a complex with PTPN22, a phosphatase overexpressed in CLL cells which may positively regulate the AKT signal downstream of the BCR pathway. As tonic BCR signal acts principally to activate AKT, results from this study suggest that HSP90 may regulate the PI3K/AKT signaling axis in CLL cells at multiple points via stabilization of: (i) BCAP protein which recruits PI3Kδ to the BCR signalosome, (ii) AKT protein, and finally, (iii) PTPN22 which also activates AKT [16]. In addition to stabilization of the BCR signal, experimental evidence indicates that HSP90 may also regulate the FGFR signal by chaperoning the constitutively active FGFR3 in CLL cells. Together, these findings point to a pivotal role of HSP90 in regulating the key survival signaling axes in CLL cells. Indeed, pharmacologic inhibition of HSP90 induces apoptosis in CLL cells by reducing the expression of MCL-1 and XIAP.

In total, accumulated evidence from this study indicates that overexpression of HSP90 may promote resistance of the leukemic B-cells to BCR-targeted agents. Thus, disruption of HSP90-chaperon activity may be an effective way to sensitize CLL cells to current therapeutic agents, particularly from those CLL patients who become unresponsive to BCR-targeted therapies.

## MATERIALS AND METHODS

### Clinical samples

All CLL patients provided written informed consent according to the Declaration of Helsinki to the University of Oklahoma Health Sciences Center (OUHSC) Institutional Review Board, which approved these studies. Primary CLL cells were isolated and purified from blood of previously untreated CLL patients at or near diagnosis (*n* = 19; clinical features are shown in Supplementary Table 1) using RosetteSep B-cell enrichment kit (STEMCELL Technologies). CLL patients were chosen randomly independent of their prognostic factors however, previously treated patients were excluded from the study. The typical purification range of CD5^+^/CD19^+^ CLL cells for this work was >99%. Purified normal CD19^+^ peripheral B-cells (purification range: >95%–99%) from healthy, age-matched individuals (*n* = 8) were purified as described earlier [17] and included as controls wherever appropriate. Cells were cultured in serum-free AIM-V (GIBCO) medium as needed. Of note, we did not supplement fetal bovine serum (FBS) to CLL cell cultures as prior study found that FBS induces spontaneous apoptosis in CLL cells [28]; instead, we used serum-free AIM-V basal media that contain human serum albumin to support primary CLL cell growth [29].

### Reagents

A high-affinity HSP90-inhibitor, AUY922 [30] was purchased from Selleckchem. Antibodies to HSP90_αβ_, PLCγ2, BCAP, CD19, AXL, BCL2, GAPDH and actin were purchased from Santa Cruz Biotechnologies. Antibodies to CD79a, CD79b, LYN, SYK, BTK, AKT, P-ERK1/2, ERK1/2, STAT3, PTPN22, FGFR3, and MCL-1 were purchased from Cell Signaling Technologies. XIAP antibody, chromogen-conjugated antibodies to human CD5 and CD19 or fluorescein isothiocyanate (FITC)-conjugated annexin V were obtained from BD Biosciences or Invitrogen, respectively. Propidium iodide (PI) and other chemicals were purchased from Sigma or Bio-Rad. Replication-deficient lentiviral constructs expressing HSP90-specific shRNA or GFP tagged control scrambled shRNA were purchased from Santa Cruz Biotechnologies.

### Treatment of CLL cells with AUY922 and determination of apoptosis induction

Purified CLL cells (1.0 × 10^6^ cells/mL) from previously untreated CLL patients (*n* = 5) were treated with increasing doses (0.05–2.0 µM) of AUY922 for 72 hours or left untreated (DMSO) and apoptosis induction was determined by flow cytometry after staining the cells with annexin V-FITC/PI as described earlier. As needed, CLL cells (4.0 × 10^6^/mL) were treated with DMSO or AUY922 (0.2 µM) for 24 hours and whole cell lysates were prepared as described earlier [31] for western blot analysis (see below).

### Transduction of primary CLL cells with lentivirus shRNA constructs

CLL cells (2.5 × 10^6^ cells/mL) were transduced with a replication-deficient lentivirus construct expressing HSP90-specific *shRNA* or scrambled-*shRNA* following manufacturer’s protocol. After 24 hours, cells were washed and lysates were prepared as described previously [31].

### Western blot analysis and immunoprecipitation

Equal amount of lysates from purified CLL cells or normal B-cells were separated by Sodium Dodecyl Sulfate (SDS)-polyacrylamide gel electrophoresis (PAGE), transferred to nitrocellulose or PVDF membranes and western blot analysis was performed using specific antibodies as described earlier [31].

As needed, proteins were immunoprecipitated [17, 18] from 0.2 mg of CLL cell lysates using specific antibodies, followed by addition of Protein G Agarose Beads (Cell Signaling) for an overnight incubation at 4°C on a rotator. After wash, precipitated immune complex was analyzed for interacting proteins in western blots using specific antibodies.

## SUPPLEMENTARY MATERIALS


