# Supplementary material for: HSP90 overexpression potentiates the B-cell receptor and fibroblast growth factor receptor survival signals in chronic lymphocytic leukemia cells
Source: Oncotarget. 2020 Jun 2;11(22):2037–46. doi: 10.18632/oncotarget.27409 (PMC7275782; doi:10.18632/oncotarget.27409)
Supplement: Supplementary file 1 [file oncotarget-11-2037-s001.pdf]

HSP90 overexpression potentiates the B-cell receptor and fibroblast growth factor receptor survival signals in chronic lymphocytic leukemia cells

SUPPLEMENTARY MATERIALS

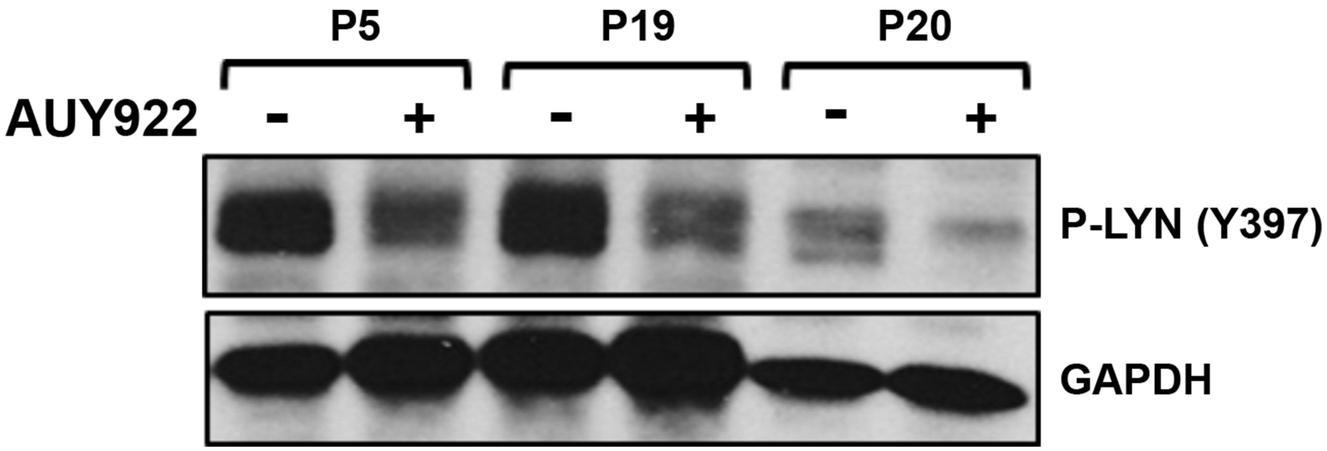

Supplementary Figure 1: HSP90 inhibition in CLL B-cells reduces phosphorylation on LYN kinase. Purified CLL B-cells obtained from previously untreated CLL patients (P5, P19, P20) were treated *in vitro* with a high-affinity HSP90-inhibitor AUY922 (0.2 µM) or DMSO (vehicle control) for 24 hours as described in the "Materials and Methods" section of the main text. Cell lysates were analyzed to detect P-LYN (Y397) status in western blot using a phospho-specific antibody. GAPDH was used as loading control.

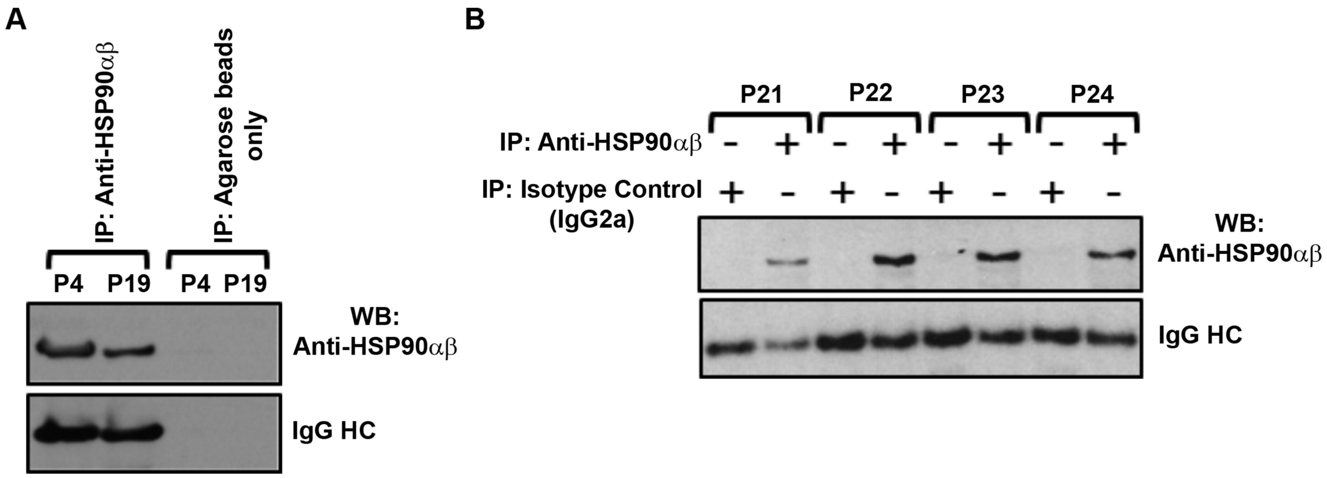

Supplementary Figure 2: Immunoprecipitation of HSP90 from CLL B-cell lysates. HSP90 was immunoprecipitated from lysates of purified CLL B-cells from previously untreated CLL patients (P4, P19, P21 – P24) using a specific antibody to $HSP90_{\alpha\beta}$, protein G conjugated to agarose beads (**A**) or isotype control antibody (IgG2a) (**B**). The immunecomplexes were analyzed for the presence of HSP90 in western blots. IgG heavy chain (HC) was used as loading control.

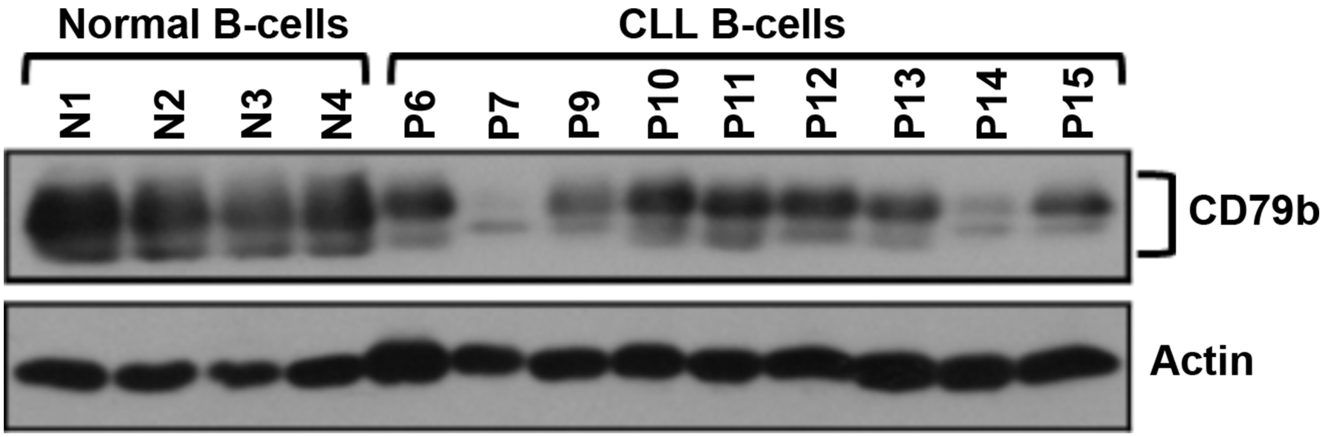

Supplementary Figure 3: CLL B-cells express reduced levels of CD79b. Lysates from purified normal B-cells (N1 – N4) and CLL B-cells from previously untreated CLL patients (P6, P7, P9, P10 – P15) used in Figure 1C were also analyzed for the expression of CD79b in western blot using a specific antibody. Actin was used as loading control.

Supplementary Table 1: Prognostic information of the CLL patients whose leukemic B-cells were used in this study

Patient No.	Rai-stage	FISH	IGHV Mutation	ZAP70 (>20%)	CD38 (>30%)
P1	I	Trisomy 12 & t(14:18)	UM-IGHV	+	+
P2	0	13q-	N/A	N/A	N/A
P3	0	Normal	N/A	N/A	N/A
P4	IV	Trisomy 12,18,19	N/A	N/A	N/A
P5	I	Normal	N/A	N/A	–
P6	0	Normal	UM-IGHV	–	–
P7	II	13q-	M-IGHV	–	–
P8	III	13q-	M-IGHV	–	–
P9	0	13q-	M-IGHV	–	–
P10	IV	13q-	N/A	–	–
P11	I	13q-/17p-	UM-IGHV	+	+
P12	II	13q -	N/A	–	+
P13	0	13q -	UM-IGHV	+	–
P14	IV	13q -	N/A	–	–
P15	0	13q -	M-IGHV	–	–
P16	0	13q -	N/A	N/A	–
P17	I	N/A	N/A	+	+
P18	I	Trisomy12 & 13q-	M-IGHV	+	+
P19	N/A	N/A	UM-IGHV	–	–

N/A: Not available.
